# POU6F1 promotes ferroptosis by increasing lncRNA-CASC2 transcription to regulate SOCS2/SLC7A11 signaling in gastric cancer

**DOI:** 10.1007/s10565-024-09843-y

**Published:** 2024-01-25

**Authors:** Jingyun Wang, Qiaoyu Jia, Shuqin Jiang, Wenquan Lu, Hanbing Ning

**Affiliations:** 1 Department of Gastroenterology, The First Affiliated Hospital of Zhengzhou University, No.1 Jianshe East Road, Erqi District, Zhengzhou, Henan 450000 People’s Republic of China; 2 Department of Child Development and Behavior, The Third Affiliated Hospital of Zhengzhou University, Zhengzhou, Henan 450000 People’s Republic of China; 3 Department of Gastroenterology, The Second Affiliated Hospital of Zhengzhou University, No.2 JingBa Road, Jinshui District, Zhengzhou, Henan 450014 People’s Republic of China

**Keywords:** Transcription factor POU6F1, LncRNA-CASC2, SOCS2, Ferroptosis, Gastric cancer

## Abstract

**Objective:**

This study investigated the effect and mechanism of POU6F1 and lncRNA-CASC2 on ferroptosis of gastric cancer (GC) cells.

**Methods:**

GC cells treated with erastin and RSL3 were detected for ferroptosis, reactive oxygen species (ROS) level, and cell viability. The expression levels of POU6F1, lncRNA-CASC2, SOCS2, and ferroptosis-related molecules (GPX4 and SLC7A11) were also measured. The regulations among POU6F1, lncRNA-CASC2, FMR1, SOCS2, and SLC7A11 were determined. Subcutaneous tumor models were established, in which the expressions of Ki-67, SOCS2, and GPX4 were detected by immunohistochemistry.

**Results:**

GC patients with decreased expressions of POU6F1 and lncRNA-CASC2 had lower survival rate. Overexpression of POU6F1 or lncRNA-CASC2 decreased cell proliferation and GSH levels in GC cells, in addition to increasing total iron, Fe2+, MDA, and ROS levels. POU6F1 directly binds to the lncRNA-CASC2 promoter to promote its transcription. LncRNA-CASC2 can target FMR1 and increase SOCS2 mRNA stability to promote SLC7A11 ubiquitination degradation and activate ferroptosis signaling. Knockdown of SOCS2 inhibited the ferroptosis sensitivity of GC cells and reversed the effects of POU6F1 and lncRNA-CASC2 overexpression on ferroptosis in GC cells.

**Conclusion:**

Transcription factor POU6F1 binds directly to the lncRNA-CASC2 promoter to promote its expression, while upregulated lncRNA-CASC2 increases SOCS2 stability and expression by targeting FMR1, thereby inhibiting SLC7A11 signaling to promote ferroptosis in GC cells and inhibit GC progression.

**Supplementary Information:**

The online version contains supplementary material available at 10.1007/s10565-024-09843-y.

## Introduction

Gastric cancer (GC) remains a globally lethal and fifth most diagnosed cancer (Sung et al. 2021). *H. pylori* infection represents one of the most well-described hazard for non-cardia GC, and its chronic infection in the gastric mucosa results in gradual development from atrophic gastritis and intestinal metaplasia (Smyth et al. 2020). Approaches are being pursued to restrain the progression of GC, such as *H. pylori* eradication (Sexton et al. 2020). Although improved chemotherapy and surgical treatment are effective, the prognosis of these patients remains unfavorable (Alsina et al. 2023). Hence, uncovering the molecules involved in the pathogenesis of GC is important for developing specialized therapies for these patients.

Ferroptosis is typically characterized by massive iron accumulation and lipid peroxidation (Li et al. 2020). Iron is one of the important nutrients for humans, while iron oxidation can lead to tumorigenesis and tumor progression, particularly in GC (Gu et al. 2022; Wang et al. 2022). The activation of ferroptosis gives rise to cell death, thus delaying the progression of GC and colorectal cancer (Xu et al. 2021). The functions of ferroptosis and ferroptosis-associated non-coding RNAs (ncRNAs) in the initiation of GC have attracted much attention recently (Lu et al. 2022; Wei et al. 2021). LncRNAs are a diverse class of RNAs that are widely involved in biological processes and perform key roles in chromatin remodeling, transcriptional regulation, and post-transcriptional modification *via* diverse chromatin-targeted mechanisms and interactions with other RNA species (Dykes and Emanueli 2017; Schmitz et al. 2016). Several studies have demonstrated that lncRNA-cancer susceptibility 2 (CASC2) acts as a tumor-suppressive lncRNA in GC (Li et al. 2016; Li et al. 2019; Zhou et al. 2017), but whether lncRNA-CASC2 can mediate ferroptosis in GC remains unknown, and the specific mechanisms are still largely to be defined.

It has been reported that SOCS2 may aggravate mitochondrial dysfunction and accelerate ferroptosis in pancreatic cancer cells (He et al. 2023). Additionally, SOCS2 can promote the K48-linked ubiquitination and degradation of SLC7A11 to facilitate ferroptosis of hepatocellular carcinoma (HCC) cells, thus affecting the radiosensitivity and prognosis of this cancer (Chen et al. 2023). A recent study has reported lncRNA-CASC2-induced positive regulation of SOCS2 in the setting of diabetic nephropathy (Min and Xie 2020). However, whether this relationship also functioned to regulate ferroptosis in GC remains unknown.

The transcription of ncRNAs, including lncRNAs, can be mediated by multiple core transcription factors (Wu et al. 2014). POU class 6 homeobox 1 (POU6F1) participates in transcriptional regulation, which is closely associated with tumor stage and mortality of lung adenocarcinoma (McClard et al. 2018; Xiao et al. 2022). Through the JASPAR database, we found two binding sites between POU6F1 and the lncRNA-CASC2 promoter. Based on these findings, the present study planned to verify the possible interactions among POU6F1, lncRNA-CASC2, and ferroptosis in GC.

## Materials and methods

### Obtaining on clinical tissues

Based on Response Evaluation Criteria in Solid Tumors (RECIST), tumor and their corresponding precancerous tissues from 40 GC patients were selected from sample bank in the First Affiliated Hospital of Zhengzhou University. Immediately after surgical resection, the tissues were frozen and stored in liquid nitrogen.

Participants meeting following criteria were eligible for inclusion: 18–75 year old; GC diagnosed with histological evidence; without history of chemotherapy or radiotherapy; and free from other serious diseases (except GC).

Participants meeting following criteria were excluded: more than one cancer; significant multimorbidities; unwilling to participate; incomplete medical information or lost in follow-up; and pregnant or breastfeeding women.

### Cell culture

Human GC cell lines include SNU719, AGS, MKN-45, and HGC-27. Human normal gastric epithelial cell line (GES-1) was used as control. All cells were purchased from ATCC (Manassas, Virginia, USA) and cultured in RPMI-1640 medium (Gibco, Grand Island, NY, USA) containing 10% FBS, 1% penicillin, and 1% streptomycin. Ferroptosis in GC cells were induced by 10 μM erastin (S7242, Selleck, TX, USA) and 2 μM RSL3 (S8155, Selleck) for 48 h.

### Cell transfection

The lentiviral overexpression plasmid of POU6F1/CASC2 and the lentiviral overexpression/knockdown plasmid of SOCS2 were synthesized by WZ Biosciences Inc (Shandong, China).

The overexpression lentiviral plasmids were transfected into 293T cells via liposomes for lentiviral packaging. AGS and MKN-45 cells were infected (multiplicity of infection: 10), and puromycin was used to screen cells with stable overexpression. Knockdown plasmids were transfected by liposomes using LipoFiterTM transfection reagent (Hanbio, Shanghai, China) in AGS and MKN-45 cells. Transfection in each group was set up in three replicates. The overexpression plasmid vector was pCDH-GFP+puro-3xFlag (negative control: vector); the knockdown plasmid vector was sh-RNA (negative control: sh-NC). The lentiviral packaging plasmids psPAX2 and pMD2.G were purchased from Wuhan Bio-Tower (China).

### Reverse transcription quantitative polymerase chain reaction (qRT-PCR)

TRIZOL (Invitrogen, Carlsbad, CA, USA) was used for total RNA extraction. RNA of 5 μL was diluted to 1:20 with RNA enzyme-free ultrapure water to detect the observance at 260 nm and 280 nm. A reverse transcription kit (TaKaRa, Tokyo, Japan) and LightCycler 480 (Roche, Indianapolis, IN, USA) instrument were used with reaction condition of pre-denaturation at 95 °C for 10 min, denaturation at 95 °C for 10 s, annealing at 60 °C for 20 s, and extension at 72 °C for 34 s, for 40 cycles. The primer sequences are in Table 1.

**Table 1:** Primer sequences for reverse transcription quantitative polymerase chain reaction

Name of primer	Sequences
POU6F1-F	TGTCCAAGCCACATACTCCA
POU6F1-R	CAGATGGCTGACTGGCTGTA
LncRNA CASC2-F	ATTGGAGGATGAGGCAGATG
LncRNA CASC2-R	CAGCAAGATCAGAGCACAGC
SOCS2-F	GTGCAAGGATAAGCGGACAG
SOCS2-R	GTAAAGGCAGTCCCCAGATG
GAPDH-F	TCAAGAAGGTGGTGAAGCAGG
GAPDH-R	TCAAAGGTGGAGGAGTGGGT

*F*, forward; *R*, reverse

### Western blot

Clinical tissue or tumor tissues from nude mouse were ground in liquid nitrogen. GC cells were lysed and placed on ice for 30 min for centrifugation at 4 °C and 12,000 rpm for 10 min. Then a 0.5-mL centrifuge tube was used to aspirate the supernatant for storage at −80 °C. After protein was mixing with loading buffer for boiling water bath to denature the protein, the proteins were electrophoresed on a 60 V and 120 V electrode. The proteins in PVDF membrane were treated with 5% skimmed milk-TBST for incubation for 1~2 h and treated with antibodies of POU6F1 (ab30944), SOCS2 (ab109245), GPX4 (ab125066), SLC7A11 (ab175186), and GAPDH (ab8245) in an incubator for overnight at 4 °C. All antibodies were from Abcam with a diluted ratio of 1:1000. After TBST washing (3 × 10 min), the proteins were incubated with HRP labeled goat anti rabbit IgG (1:5000, CWBIO, Beijing, China) for 1 h, followed by TBST washing (3 × 10 min). The membranes were subjected to color development and data analysis.

### CCK-8 assay

Cell suspension (100 μL, 1×10^5^ cells/ml) were inoculated in each well of the 96-well plates. The volume of CCK-8 reagent (Dojindo) was 10 μL. After cell reaction for 24 h, the optic value was measured at 450 nm.

### Measurement on total iron, lipid peroxidation (MDA), and glutathione (GSH) levels

The levels of total iron (OD at 593 nm) and Fe^2+^ (OD at 593 nm) were measured in GC cells using an iron assay kit (ab83366, Abcam). MDA production (OD at 532 nm) was measured using (MDA) Kit (ab233471, Abcam). GSH levels (OD at 405 nm) were measured using GSH Kit (ab239709, Abcam). The above assays were performed using a FlexStation 3 multifunctional enzyme marker (Flexstation3, Molecular Devices, Sunnyvale, USA).

### Reactive oxygen species (ROS) levels using flow cytometry

AGS and MKN-45 cells were centrifuged (1200 rpm, 5 min) to collect cells. ROS levels were determined using a ROS kit (S0033S, Beyotime). The DCFH-DA was diluted with serum freed medium at 1:1000 to reach a final concentration of 10 μmol/l. Then 1 ml of DCFH-DA was added into cells for incubation at 37 °C for 20 min and blending every 3 min. Cells were washed (× 3 times) and detected using cytometry (CytoFLEX, BECKMAN, CA, USA).

### Dual luciferase reporter gene assay

The binding sites for POU6F1 on the lncRNA-CASC2 promoter were predicted on JASPAR (http://jaspar.genereg.net/). The sequences of the binding sites (site 1 and site 2) were designed and synthesized for inserting into the luciferase reporter gene vector (pGL3-Basic, Promega, Madison, WI, USA) and cell transfection. The fluorescence intensity was measured using a dual luciferase reporter gene assay kit (Promega).

### Chromatin immunoprecipitation quantitative PCR (ChIP-qPCR)

SimpleChIP Plus sonication chromatin IP kit (Cell Signaling Technology) was used. GC cells were fixed in 1% formaldehyde for DNA and protein cross-linking. Chromatin was cut using Microson Ultrasonic Cell DisruptorXL (Misonix) and was reacted with antibodies (anti-POU6F1 and IgG antibody) to enable the immunoprecipitates bind protein G magnetic beads. Then Protein-DNA cross-linking was reversed, and DNA was purified, in which the enrichment of CASC2 promoter was detected.

### RNA pull-down assay

Pierce TMMagnetic RNA-Protein Pull-Down kit (Millipore, Billerica, MA, USA) was applied. Biotinylated lncRNA CASC2 (Geneseed, Guangzhou, China) or biotinylated NC were incubated with GC cell lysate. The complexes were captured with streptavidin-labeled immunomagnetic beads and then incubated with proteinase K-contained buffer at 25 °C for 1 h.

### RIP assay

Cells were treated with RIP lysate for preparation of cell lysate. The re-suspended beads were reacted with FMR1 antibody (5 μg, ab259335, 1:100, Abcam). Then beads added RIP Wash Buffer (500 ul) for vortex and shaking, with the supernatant being discarded. This centrifugation and washing were repeated for one more time. The magnetic beads were added RIP immunoprecipitation buffer (900 ul). The pre-thawed cell lysis was centrifuged for 10 min (14,000 rpm, 4 °C), from which 100 ul supernatant was reacted with complex. After above centrifugation and supernatant removal process again, the tube was added RIP wash buffer (500 ul) again with the supernatant being removed. After washing for six times, the tube was re-suspended with bead-antibody complex in Proteinase K buffer (150 μl) (55 °C, 30 min). After supernatant in the tube was removed, the RNA was extracted for qRT-PCR.

### Immunoprecipitation (Co-IP)

Cells were lysed in IP buffer (150 mM NaCl, 10 mM Tris-HCl, pH 7.5, 1% Nonidet P-40, supplemented with 1% protease inhibitor) for 30 min at 4 °C and treated with 20 mM NEM (N-ethylmaleimide) for centrifugation (12,000 rpm, 10 min, 4 °C), from which the supernatant was collected and reacted with SOCS2 or SLC7A11 antibodies for 2 h at 4 °C, followed by 2 h of incubation with ProteinA/G agarose beads at 4 °C. After cold IP buffer washing for five times, the complexes were collected by centrifugation (2000 rpm, 2 min) and boiled with 2×SDS sample buffer before western blot.

### mRNA Stability

The expression of SOCS2 mRNA in Actinomycin D (1 μg/ml, Sigma-Aldrich)-treated GC cells at 0 h, 1 h, 4 h, 8 h, 12 h, and 24 h was detected by qRT-PCR.

### Subcutaneous tumor formation in nude mouse

All experiments were conducted according to the regulation of experimental animals of the First Affiliated Hospital of Zhengzhou University (Approval no. 202310107). SPF staged BALB/c nude mice (*n*=24, 4–6 weeks old, 16 ± 2 g) from Hunan SJA Laboratory Animal Co., Ltd. were kept in a sterile laminar flow room at regular housing condition. Nude mice were randomly divided into four groups and received sodium pentobarbital (30 mg/kg) for anesthesia. MKN-45 cells stably expressing POU6F1 and CASC2 (2×10^6^ in 0.1 mL PBS) were injected subcutaneously into the left side of mice. When the tumor volume reached approximately > 60 nm^3^, 15 mg/kg of erastin was injected. The tumor volume was calculated at a regular base. After 5 weeks, mice were sacrificed and the tumor volume was calculated: 1/2 × long diameter × short diameter^2^. The tumor tissue was paraffin-embedded and sectioned for immunohistochemistry (IHC).

### IHC

The paraffin sections (4 μm) were baked and then routinely dewaxed before 3% H_2_O_2_ was added and reacted. The slices were blocked with normal goat serum. After the excessive liquid was removed, primary antibodies of Ki-67 (12202S, 1:400), SLC7A11 (98051 , 1:400, cell signaling, Boston, USA), and SOCS2 (ab247806, 1:500, Abcam) were added, and the slices were reacted with secondary antibody for 1 h, followed DAB coloring and re-staining with hematoxylin. After dehydration and transparency, the slices were sealed for observation.

### Statistical analysis

Data were processed using GraphPad prism7 and presented as mean ± standard deviation (mean ± SD). *T* test and one-way analysis of variance test were respectively used for pairwise comparison or comparison among multiple groups, with Tukey’s multiple comparisons test for post hoc comparisons. Kaplan-Meier survival analysis and Pearson correlation were performed to detect the association of target proteins with prognosis or their correlations. *p* < 0.05 indicates significant difference.

## Results

### Poorly expressed POU6F1 and lncRNA-CASC2 are correlated with decreased survival rate of GC patients

**Fig. 1:**
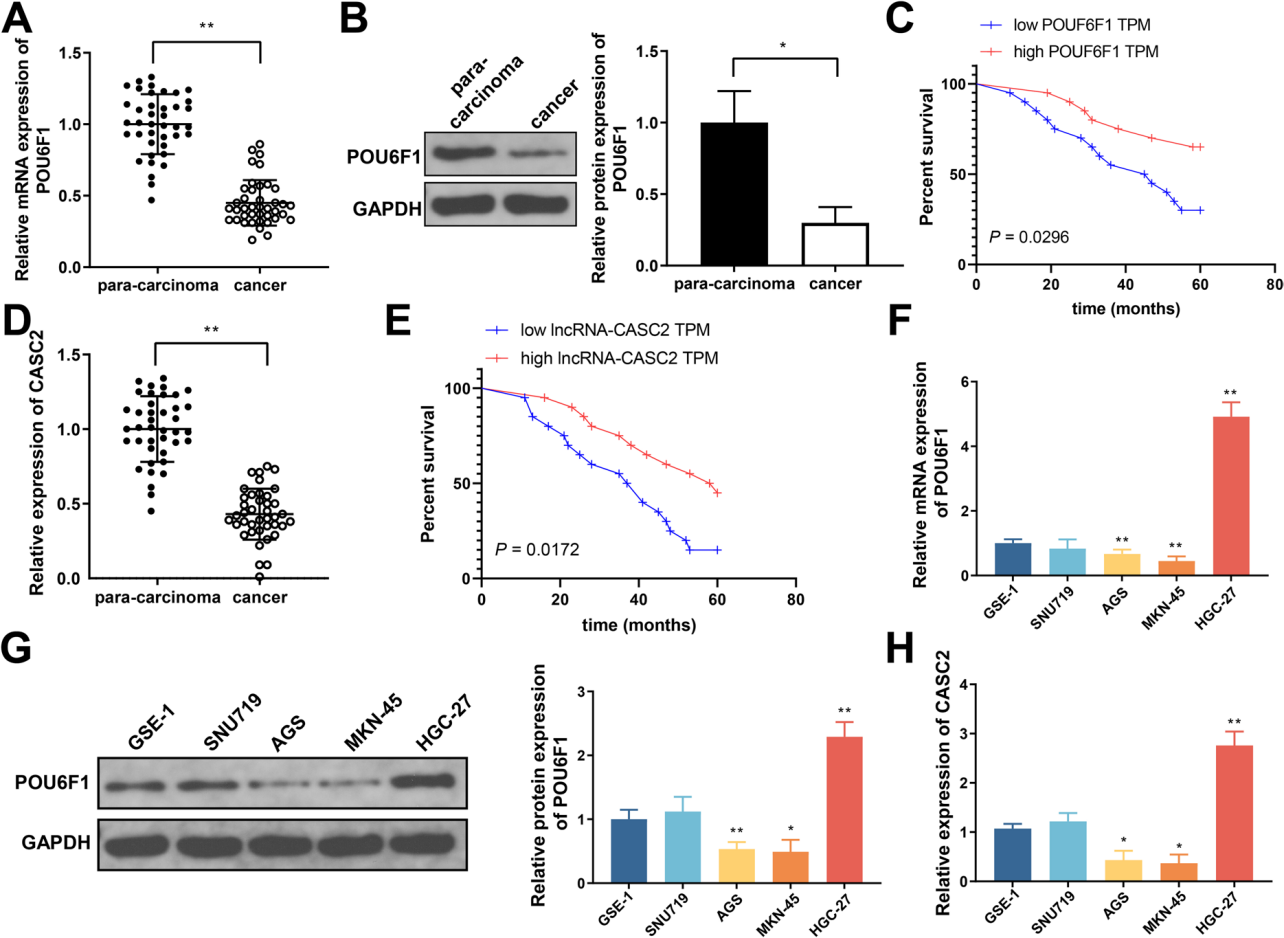
POU6F1 and lncRNA-CASC2 were lowly expressed in GC tissues and cell lines. **A**–**B** The mRNA and protein levels of POU6F1 in 40 patients with GC were detected. **C** Survival analysis of POU6F1 expression. **D** LncRNA-CASC2 level in 40 patients with GC was detected by qRT-PCR. **E** Survival analysis of lncRNA-CASC2 expression. **F**–**H** The mRNA and protein levels of POU6F1 as well as lncRNA-CASC2 expression in different GC cells and human normal gastric mucosal cells

As shown in Fig. 1A–B and D, lower POU6F1 mRNA and protein expressions, and lower lncRNA-CASC2 expression were found in GC tissues (vs. adjacent normal tissues). The lower POU6F1 or lncRNA-CASC2 expression was correlated with shorter survival period of GC patients (Fig. 1C and E). Additionally, the levels of POU6F1 and lncRNA-CASC2 were significantly reduced in GC cells (Fig. 1F–H, vs. GES-1). These results indicate that POU6F1 and lncRNA-CASC2 may participate in GC. AGS and MKN-45 cells were used for follow-up assays for having the most significantly decreased expressions of POU6F1 and lncRNA-CASC1.

### Overexpression of POU6F1 or lncRNA-CASC2 increases the sensitivity of GC cells to ferroptosis

**Fig. 2:**
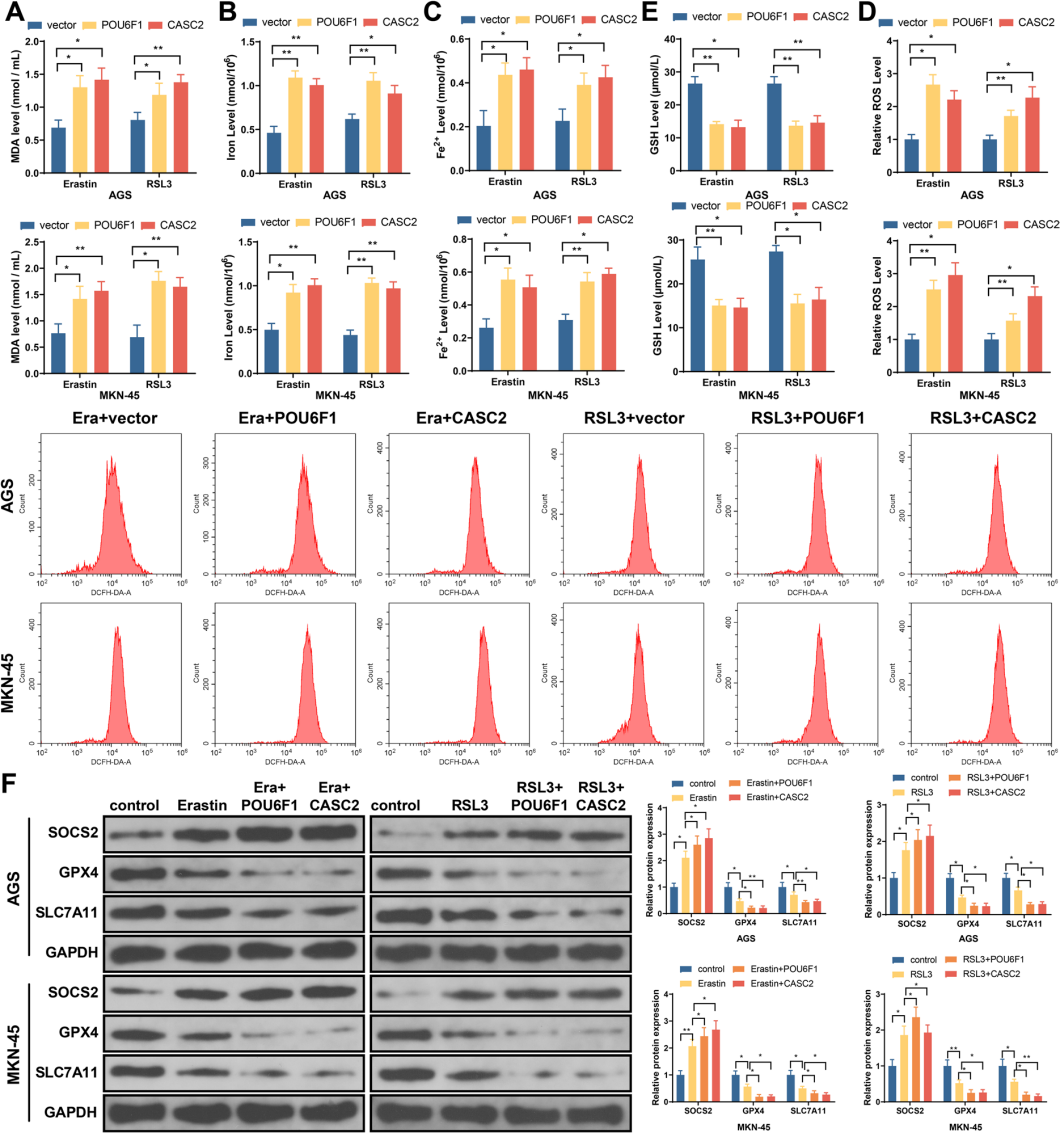
Overexpression of POU6F1 or lncRNA-CASC2 increased the sensitivity of GC cells to ferroptosis. **A** MDA level. **B** Total iron level. **C** Accumulation of Fe2^+^. **D** The ROS levels. **E** GSH levels. **F** Western blot detected SOCS2, GPX4, and SLC7A11 levels

Transfection of POU6F1 or lncRNA-CASC2 overexpression vector significantly increased the expression of POU6F1 or lncRNA-CASC2, respectively, in GC cells (Fig. SA-B). CCK-8 displayed that treatment with ferroptosis inducers erastin and RSL3 significantly inhibited the proliferation of GC cells, while overexpression of POU6F1 or lncRNA-CASC2 further increased the inhibitory effect of ferroptosis inducers on cell proliferation (Fig. SC-D). In addition, overexpression of POU6F1 or lncRNA-CASC2 led to the increase in MDA, iron, Fe2^+^, and ROS levels and the decrease in GSH levels induced by ferroptosis inducers (Fig. 2A–E). SOCS2 expression was increased, while GPX4 and SLC7A11 expressions were decreased in erastin- and RSL3-induced GC cells, while those expression patterns can be enhanced in response to the overexpression of POU6F1 or lncRNA-CASC2 (Fig. 2F). Collectively, overexpression of POU6F1 or lncRNA-CASC2 can promote ferroptosis and increase the sensitivity of GC cells to ferroptosis inducers.

### POU6F1 directly binds to lncRNA-CASC2 promoter to increase its transcriptional expression

**Fig. 3:**
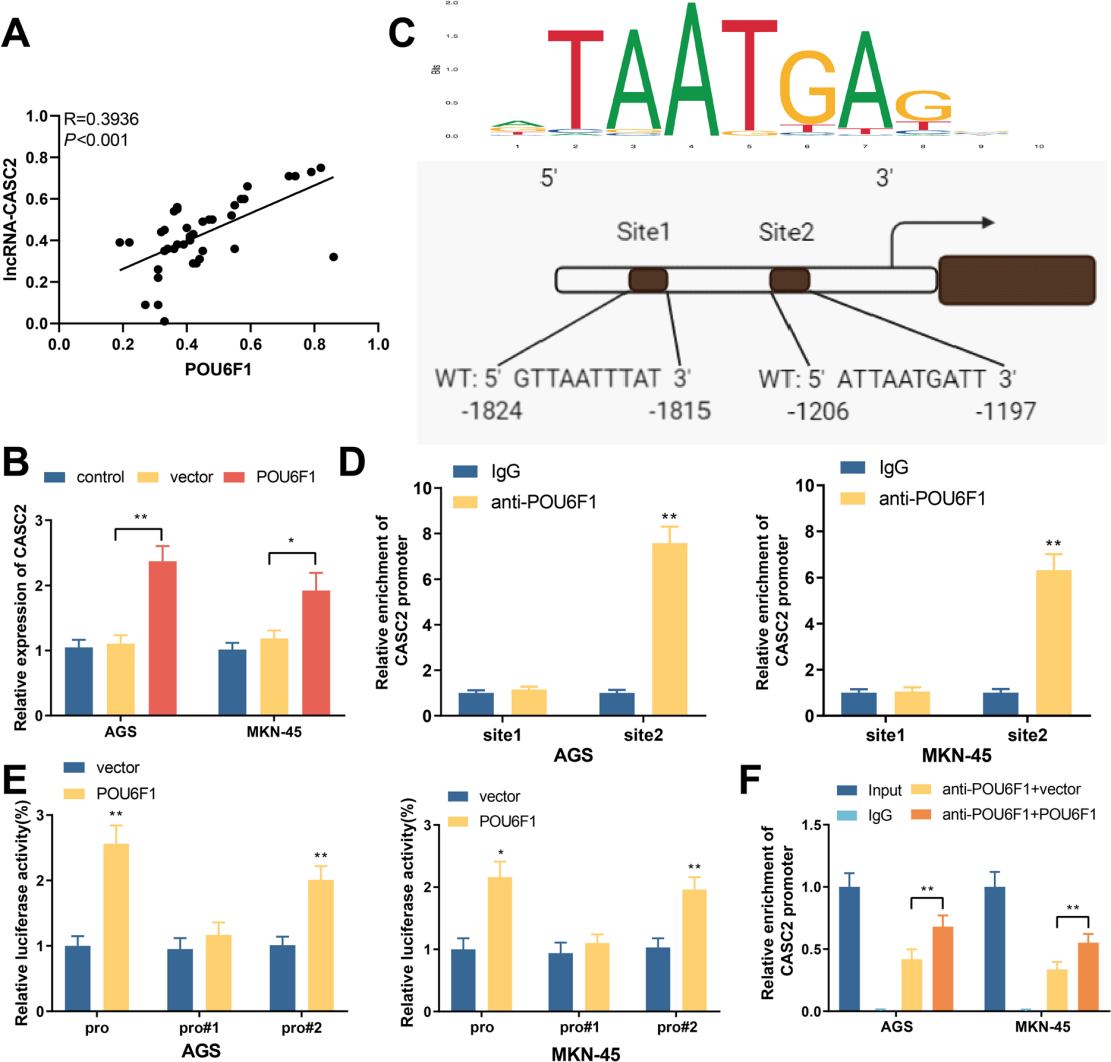
POU6F1 directly binds to lncRNA-CASC2 promoter to increase lncRNA-CASC2 transcriptional expression. **A** Correlation between clinical POU6F1 and lncRNA-CASC2 expressions analyzed by Pearson. **B** The expression of lncRNA-CASC2 in cells after cell transfection was detected by qRT-PCR. **C** The binding site of POU6F1 on lncRNA-CASC2 promoter in JASPAR database. **D** The binding relationship between POU6F1 and lncRNA-CASC2 promoters was detected by ChIP. **E** The effect of POU6F1 on transcription of lncRNA-CASC2 promoter detected by dual luciferase assay. **F** The effect of POU6F1 overexpression on its binding with lncRNA-CASC2 promoter detected by ChIP-qPCR

Pearson analysis showed a positive correlation between POU6F1 and lncRNA-CASC2 (Fig. 3A). Specifically, overexpression of POU6F1 in GC cells could increase lncRNA-CASC2, indicating that POU6F1 positively regulates the expression of lncRNA-CASC2 (Fig. 3B). Besides, as predicted by the JASPAR database, lncRNA-CASC2 promotors contained two POU6F1 binding sites (Fig. 3C). ChIP results showed that POU6F1 antibody could enrich the second binding site sequence (−1206~−1197, ATTAATGATT) on lncRNA-CASC6, but did not enrich the first binding site sequence (−1824~−1815: GTTAATTTAT) (Fig. 3D). Luciferase assay suggested that overexpression of POU6F1 could increase the transcriptional activity of lncRNA-CASC2 promoter sequence containing binding site 2, but fail to achieve any significant effect on the transcriptional activity of lncRNA-CASC2 promoter sequence containing only binding site 1 (Fig. 3E), indicating that POU6F1 increases transcriptional activity of lncRNA-CASC2 mainly by binding to the ATTAATGATT sequence of lncRNA-CASC2 promoter region. In addition, ChIP results also showed that overexpression of POU6F1 could increase the enrichment of lncRNA-CASC2 promoter by POU6F1 (Fig. 3F). These results indicate that POU6F1 can directly bind to the ATTAATGATT sequence on the lncRNA-CASC2 promoter to promote its transcriptional expression in GC cells.

### LncRNA-CASC2 knockdown can demolish the effect of POU6F1 overexpression on ferroptosis of GC cells

**Fig. 4:**
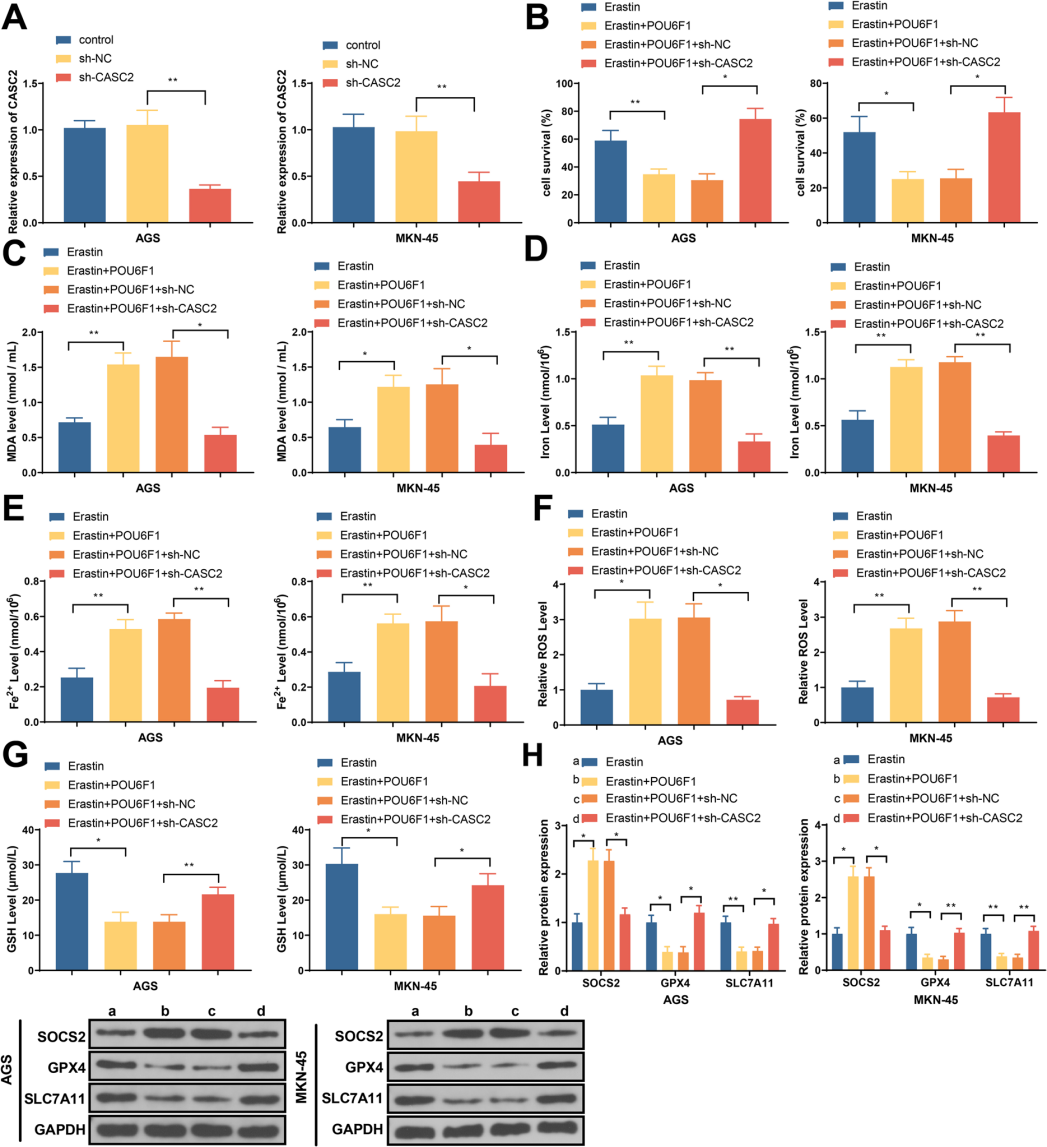
LncRNA-CASC2 knockdown can demolish the effect of POU6F1 overexpression on ferroptosis of GC cells. **A** After cell transfection, lncRNA-CASC2 expression in AGS and MKN-45 cells. Cells were treated with erastin. **B** Survival rate detected by CCK-8. **C** MDA level. **D** Total iron level. **E** Accumulation of Fe2^+^. **F** ROS level. **G** GSH levels. **H** Western blot detected the protein expressions of SOCS2, GPX4, and SLC7A11

qRT-PCR results showed GC cells transfected with lncRNA-CASC2 knockdown plasmid vector had decreased lncRNA-CASC2 expression (Fig. 4A). Overexpression of POU6F1 can enhance the inhibitory effect of erastin to proliferation of GC cells, which can be reversed by lncRNA-CASC2 knockdown, as showed by CCK-8 results (Fig. 4B). In addition to that, overexpression of POU6F1 can promote the increase of MDA, iron, Fe2^+^, ROS, and SOCS2 levels and the decrease of GSH, GPX4, and SLC7A11 induced by erastin, but such effect can be counteracted by lncRNA-CASC2 knockdown (Fig. 4C–H). Those results indicate that POU6F1 regulate ferroptosis in GC cells through mediating lncRNA-CASC2 expression.

### LncRNA-CASC2 increases SOCS2 stability and expression through FMR1

**Fig. 5:**
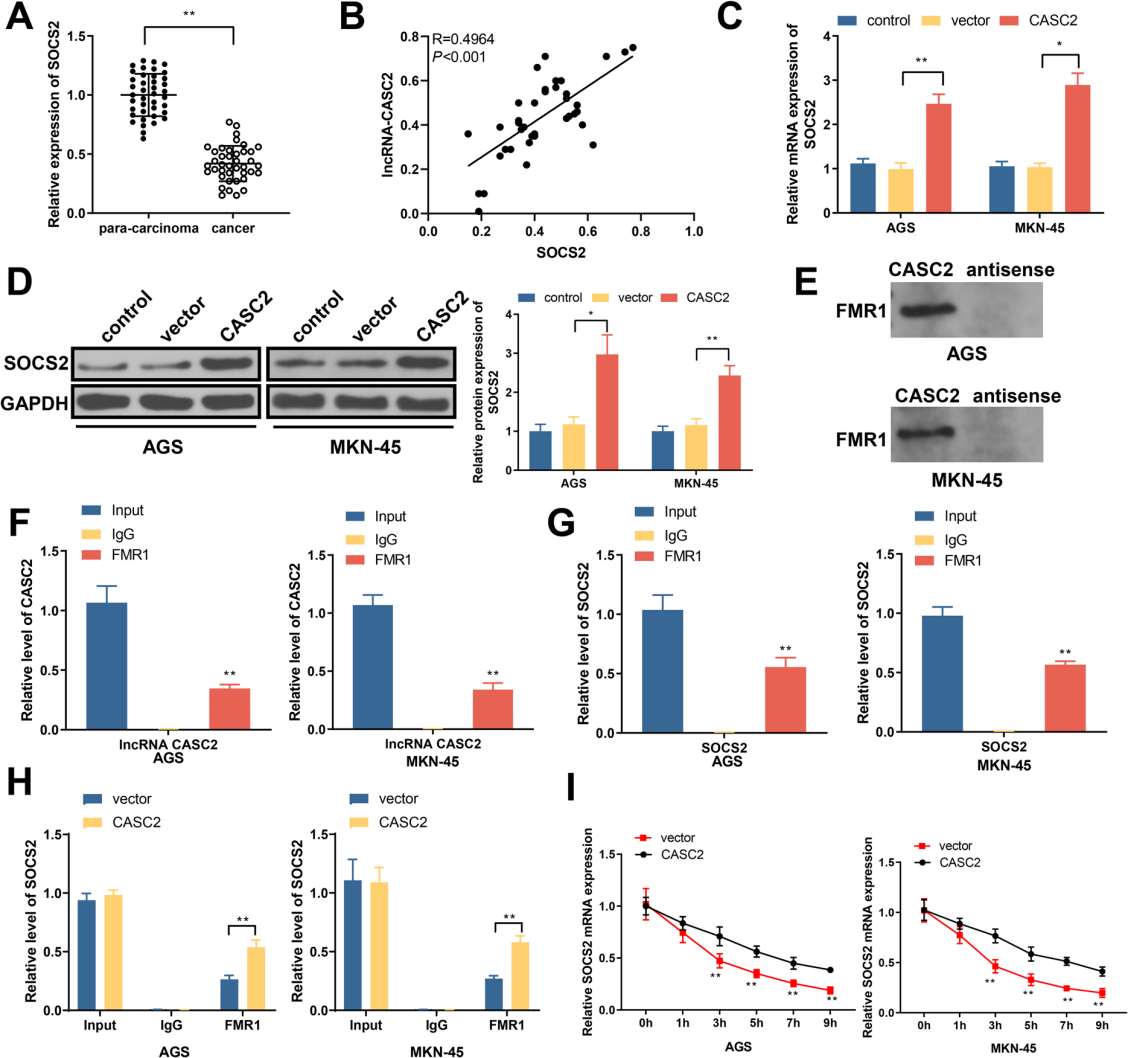
LncRNA-CASC2 can increase the binding of FMR1 with SOCS2 to promote SOCS2 stability and expression. **A** Clinical SOCS2 expression in GC tissues. **B** Correlation analysis on lncRNA-CASC2 and SOCS2 expression. **C**–**D** After cell transfection, SOCS2 mRNA, and protein expression. **E** Binding of lncRNA-CASC2 with FMR1 was detected by RNA pull down. **F** RIP verified the enrichment of FMR1 antibody to lncRNA-CASC2. **G** RIP verified the binding of FMR1 with SOCS2. **H** RIP detected the enrichment of FMR1 antibody to SOCS2 after lncRNA-CASC2 overexpression. **I** Stability of SOCS2 mRNA

The decreases of SOCS2 expression were found by qRT-PCR (Fig. 5A), which was found to be positively correlated with lncRNA-CASC2 expression in GC tissues (Fig. 5B). In addition to that, lncRNA-CASC2 overexpression can substantially increase SOCS2 mRNA and protein levels (Fig. 5C–D). RNA pull down assay showed lncRNA-CASC2 biotin probes can enrich FMR1 protein, indicating ncRNA-CASC2 can target ncRNA-CASC2 (Fig. 5E). RNA RIP demonstrated that FMR1 antibody can enrich both lncRNA-CASC2 and SOCS2, indicating the direct binding of FMR1 with lncRNA-CASC2 and SOCS2 (Fig. 5F–G). Meanwhile, lncRNA-CASC2 overexpression can enhance the enrichment of FMR1 antibody to SOCS2 (Fig. 5H), suggesting lncRNA-CASC2 can promote the binding of FMR1 with SOCS2. After actinomycin D treatment, the stability of SOCS2 mRNA was detected by qRT-PCR, which showed lncRNA-CASC2 overexpression can increase SOCS2 stability (Fig. 5I). Taken together, lncRNA-CASC2 can promote the binding of FMR1 with SOCS2 to increase SOCS2 stability and expression.

### LncRNA-CASC2 suppresses SLC7A11 expression by regulating SOCS2-mediated SLC7A11 ubiquitination degradation

**Fig. 6:**
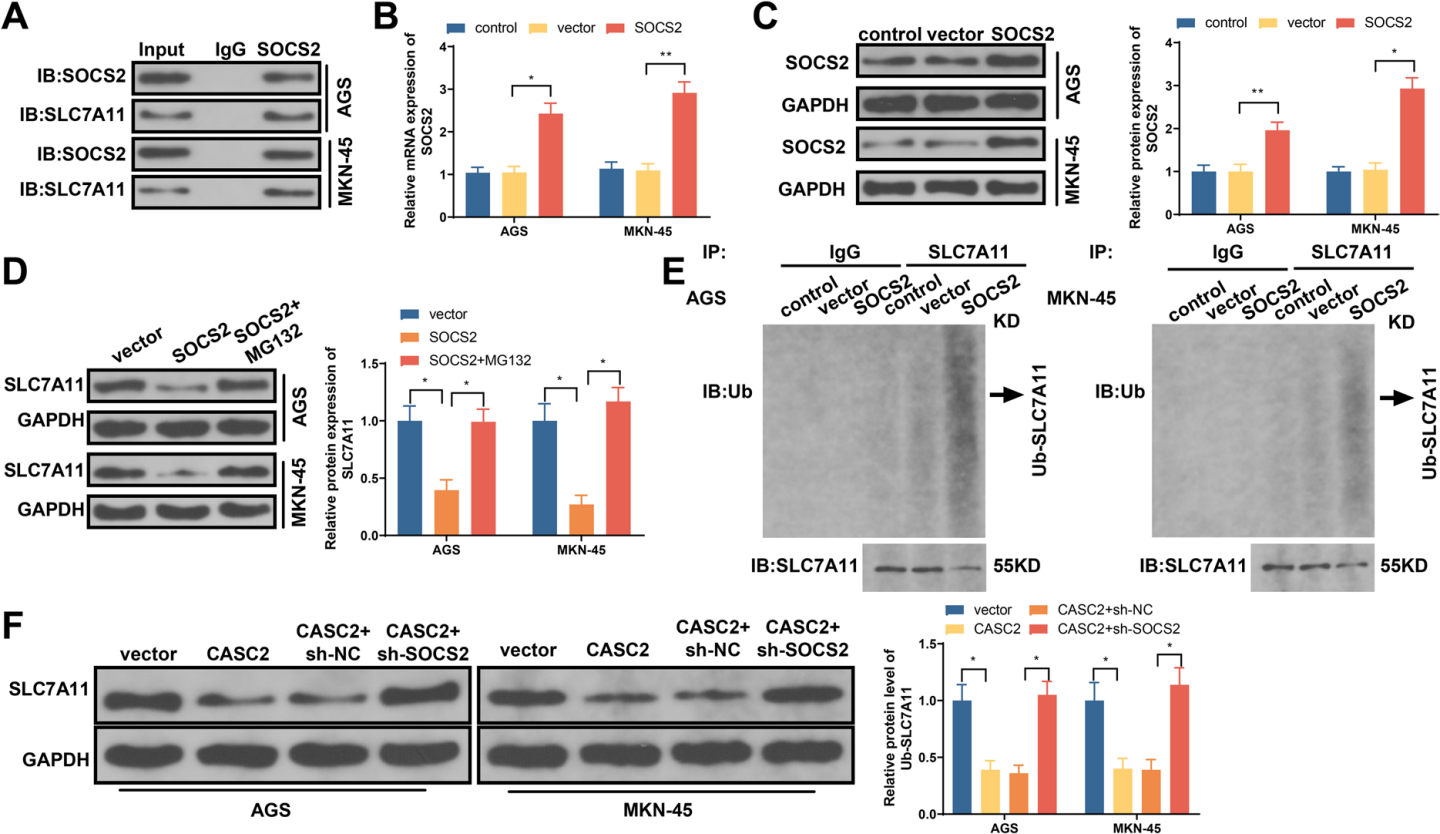
LncRNA-CASC2 mediates SOCS2 to regulate SLC7A11 ubiquitination degradation and to suppress SLC7A11 expression. **A** CoIP detected the enrichment of SOCS2 antibody to SLC7A11 protein in GC cells. **B**, **C** After cell transfection, SOCS2 mRNA, and protein expression in GC cells. **D** GC cells treated by SOCS2 overexpression and MG132, in which the expression of SLC7A11 was detected. **E** Co-IP detected the SLC7A11 ubiquitination. **F** GC cells treated by SOCS2 overexpression or sh-SOCS2, in which the expression of SLC7A11 was detected

A previous literature documented that SOCS2 can enhance SLC7A11 ubiquitination to mediate ferroptosis of hepatocellular carcinoma cells (Chen et al. 2023). The results of CoIP showed that SOCS2 antibody in GC cells can enrich SLC7A11 protein (Fig. 6A), suggesting the direct binding of SOCS2 with SLC7A11. SOCS2 overexpression vector was transfected into GC cells to elevate SOCS2 mRNA and protein expressions (Fig. 6B–C). SOCS2 overexpression can significantly suppress SLC7A11 expression, while co-treatment with proteasome inhibitors MG132 can reverse such suppression (Fig. 6D), suggesting SOCS2 regulates SLC11A7 expressions through ubiquitinated proteasomal degradation system. Co-IP further identified SOCS2 overexpression can increase SLC7A11 ubiquitination (Fig. 6E). Western blot demonstrated that lncRNA-CASC2 overexpression can suppress SLC7A11 expression in GC cells, which can be counteracted by SOCS2 knockdown (Fig. 6F). Those results concluded that SOCS2 can directly bind SLC7A11 to increase its ubiquitination degradation, while lncRNA-CASC2 in GC cells suppresses SLC7A11 expression through SOCS2.

### SOCS2 knockdown can abolish the effect of lncRNA-CASC2 overexpression on GC cell ferroptosis

**Fig. 7:**
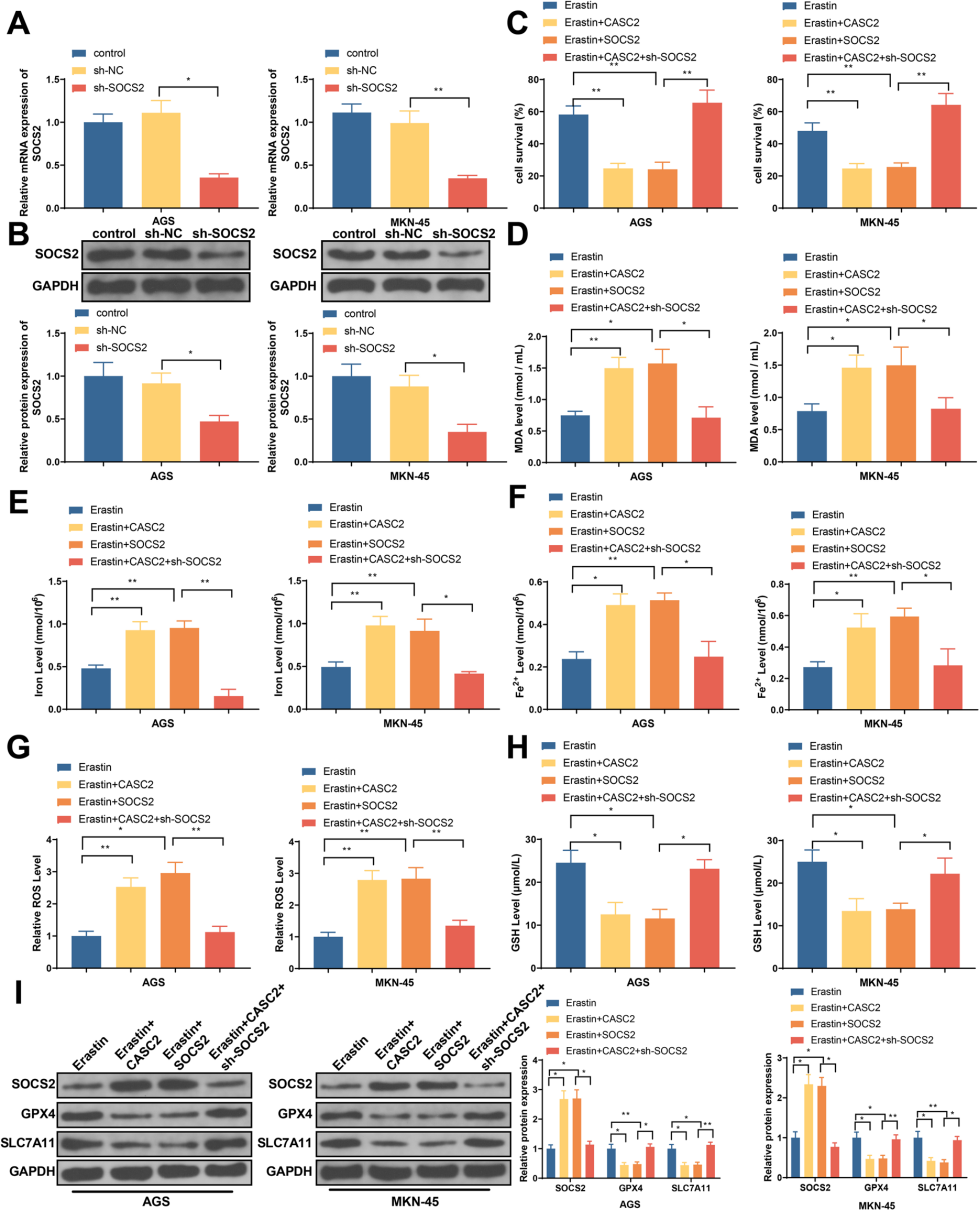
Effect of lncRNA-CASC2 overexpression on ferroptosis of GC cells can be reversed by SOCS2 knockdown. **A, B** After cell transfection, SOCS2 mRNA and protein expressions were detected. **C** Cell viability. **D** MDA level. **E** Total iron. **F** Fe2+. **G** ROS level. **H** GSH level. **I** Western blot detected SOCS2, GPX4, and SLC7A11 protein expressions

GC cells were successfully transfected with SOCS2 knockdown vector, as evidenced by suppressed mRNA and protein expressions of SOCS2 in GC cells (Fig. 7A–B). Cell viability measurement presented that overexpression of SOCS2 or lncRNA-CASC2 can promote the suppressive effect of erastin to GC cell proliferation, while SOCS2 knockdown can abolish the effect of lncRNA-CASC2 overexpression on GC cell proliferation (Fig. 7C). Erastin can induce the increase of MDA, iron, Fe2+, and ROS level, and the decrease of GSH level, which can be enhanced by SOCS2 or lncRNA-CASC2 overexpression. Meanwhile, such enhancement can be reversed by co-transfection of SOCS2 knockdown and lncRNA-CASC2 overexpression (Fig. 7D–H). The increase of SOCS2 and decrease of GPX4 and SLC7A11 levels induced by erastin can be promoted by SOCS2 or lncRNA-CASC2 overexpression, but can also be demolished by co-transfection of SOCS2 knockdown and lncRNA-CASC2 overexpression (Fig. 7I). Those results indicate that lncRNA-CASC2 can activate SOCS2 signal pathway to suppress ferroptosis of GC cells.

### POU6F1 or lncRNA-CASC2 overexpression can activate SOCS2 to promote ferroptosis and suppress tumor growth in nude mouse

**Fig. 8:**
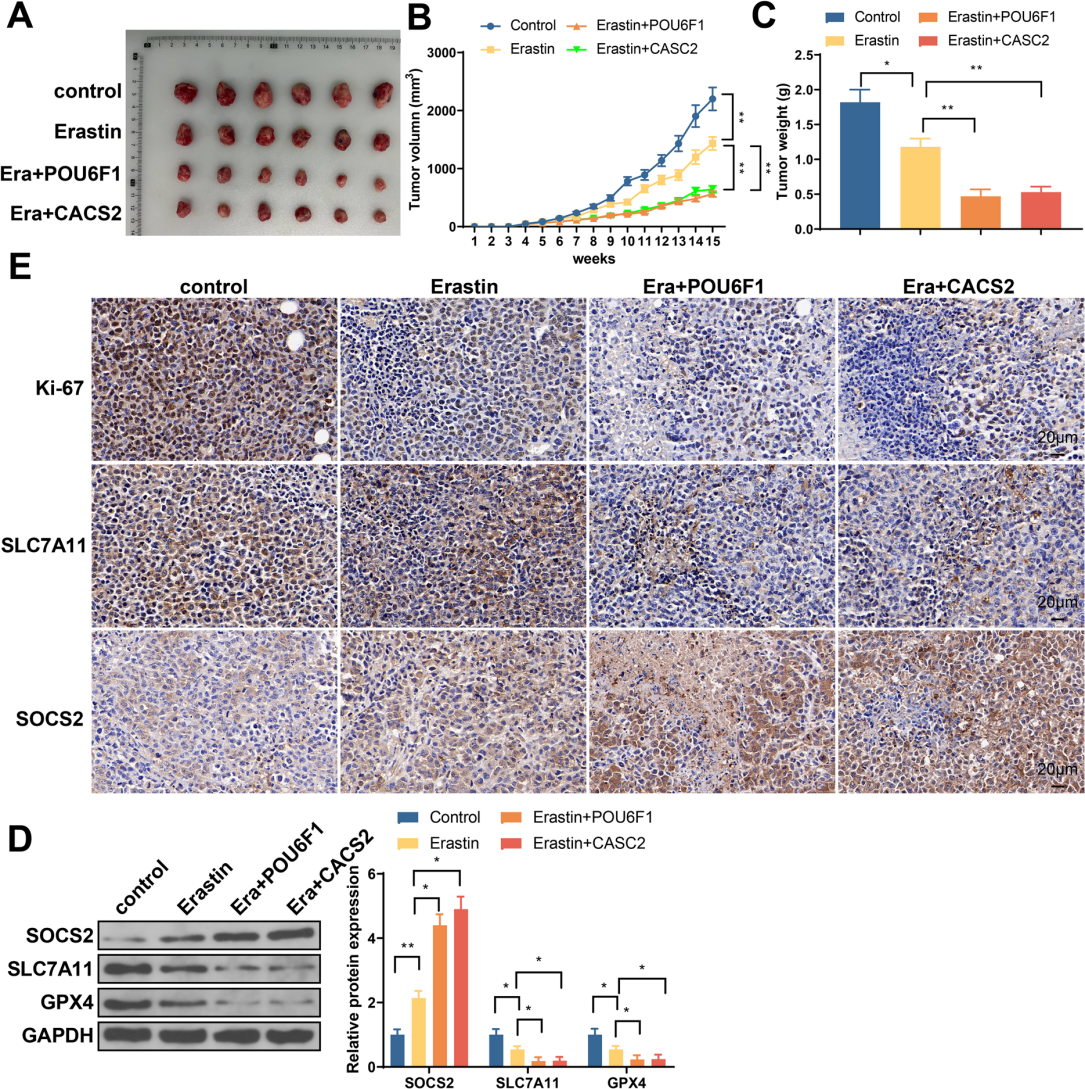
POU6F1 or lncRNA-CASC2 overexpression can activate SOCS2 to promote ferroptosis and suppress tumor growth in nude mouse. **A** Tumor extracted from nude mouse. **B** Tumor volume changes. **C** Tumor weight changes. **D** Western blot detected the expressions of SOCS2, SLC7A11, and GPX4 in tumor tissues. **E** Immunohistochemistry detected the expressions of Ki67, SOCS2, and SLC7A11 in tumor tissues

The results in Fig. 8A–C showed that erastin can suppress tumor growth, tumor volume, and weight in nude mouse, and such suppression can be enhanced by POU6F1 or lncRNA-CASC2 overexpression. Western blot results in tumor tissues showed that erastin can increase the expression of SOCS2, but inhibit SLC7A11 and GPX4 expression, which can be further promoted by POU6F1 or lncRNA-CASC2 overexpression (Fig. 8D). IHC demonstrated that Ki-67 and SLC7A11 expression in tumor tissues were decreased in response to erastin treatment, but SOCS2 expression was elevated. Such expression pattern can be further enhanced in response to cells with POU6F1 or lncRNA-CASC2 overexpression (Fig. 8E). Altogether, POU6F1 or lncRNA-CASC2 can activate SOCS2 signaling to promote ferroptosis and further inhibit tumor growth in nude mouse.

## Discussion

LncRNAs can function as modulators of gene expression at genomic, transcriptomic, or post-transcriptomic levels, contributing to their potential as biomarkers for GC (Ghafouri-Fard and Taheri 2020). Our experiments in current study demonstrated that lncRNA-CASC2 could accelerate ferroptosis and repress the growth of GC *via* mediating SOCS2-dependent ubiquitination. Moreover, we identified POU6F1 to be a positive regulator of lncRNA-CASC2 transcription, which provided an understanding of lncRNA-related mechanisms in the growth and progression of GC.

In both GC tissues and cell lines, lncRNA-CASC2 was determined to be a downregulated lncRNA. LncRNA-CASC2 has been widely demonstrated as an anti-tumor lncRNA in gastrointestinal tumors. For instance, lncRNA-CASC2 has shown growth-inhibitory and pro-apoptotic impacts on HCC cells (Fan et al. 2018). Elevation of CASC2 expression can impair the proliferation of colorectal cancer (CRC) cells and suppress tumor growth *in vivo* (Huang et al. 2016). Additionally, lncRNA-CASC2 exerts pro-apoptotic and pro-autophagic effects on human colon cancer cells (Ju et al. 2020). Similarly, it has been reported that lncRNA CASC2 is lowly expressed in colon cancer cell lines HT29 and SW480, while its re-expression restrains cancer cell viability but accelerates apoptosis and autophagy (Zhang et al. 2021). More recently, lncRNA-CASC2 upregulation impedes the proliferative, migrating, and invading behaviors of CRC cells (Kang et al. 2022). Moreover, the tumor-suppressive role of lncRNA-CASC2 has been recognized in both *in vitro* and *in vivo*. LncRNA-CASC2 represses the invading and angiogenic abilities of two GC cells SGC7901 and MGC803 while strengthening their apoptotic ability (Zhou et al. 2017). Another study has revealed that lncRNA-CASC2 in SGC-7901 and BGC-823 has the ability to suppress the cell viability and enhance the apoptosis (Li et al. 2016). In a more recent study which investigated GC, lncRNA-CASC2 has been documented to be under-expressed (Li et al. 2019), which coincides with results demonstrated in this study. However, there is no literature reporting the relationship between lncRNA-CASC2 and ferroptosis. In this study, upregulation of lncRNA-CASC2 expression was found to boost ferroptosis in GC cells and enhance their sensitivity to ferroptosis inducer erastin and RSL3, corresponding to increased contents of MDA, iron, Fe^2+^, and ROS as well as reduced GSH levels. Ferroptosis is a ROS-dependent cell death form; erastin and RSL3 are two classical ferroptosis activators that enhance the intracellular iron generation and disrupt the antioxidant system (Tang et al. 2021). The specific mechanism by which lncRNA-CASC2 mediates the levels of the aforementioned ferroptosis-related markers remains unknown, which was further discussed in our study.

In this study, POU6F1 binds to the lncRNA-CASC2 promoter and increases the transcription of lncRNA-CASC2 via binding to ATTAATGATT sequence on the lncRNA-CASC2 promoter. We experimentally determined that POU6F1 overexpression could expedite ferroptosis and strengthen the inhibitory effect of erastin on GC cells, which could be counteracted by lncRNA-CASC2 knockdown. Although POU6F1 has been unveiled to suppress the growth of lung adenocarcinoma cells (Xiao et al. 2022), there is no study showing the relationship between POU6F1 and ferroptosis. This study for the first time reported the regulation of lncRNA-CASC2 by POU6F1 and the suppressive role of POU6F1 in ferroptosis. Furthermore, this study revealed that lncRNA-CASC2 could recruit FMR1 to enhance the stability of SOCS2. FMR1 belongs to RNA-binding proteins that can precisely mediate the expression of genes from the aspects of alternative splicing, mRNA trafficking, mRNA stability, and mRNA translation (Prashad and Gopal 2021). FMR1 antibody was found in the present study to bind to both lncRNA-CASC2 and SOCS2, and lncRNA-CASC2 significantly enhanced the binding of FMR1 to SOCS2, thus upregulating the expression of SOCS2. SOCS2 can function as a bridge to transmit ubiquitin and degrade SLC7A11 by enhancing K48 polyubiquitination, which contributes to ferroptosis in HCC cells (Chen et al. 2023). In this study, CoIP results demonstrated that SOCS2 antibody could recruit SLC7A11 protein and elevate the ubiquitination level of SLC7A11. At present, SLC7A11, a cystine-glutamate antiporter, has been widely linked to ferroptosis and regarded as a ferroptosis marker (Chen et al. 2021; Fang et al. 2020; Liu et al. 2020) SLC7A11 inhibition can lead to reduced biosynthesis of glutathione, promoting lipid peroxidation and ferroptosis (Hong et al. 2021). Reversely, overexpression of SLC7A11 can facilitate tumorigenesis partly *via* repressing ferroptosis (Koppula et al. 2021), which provides evidence supporting our conclusion that lncRNA-CASC2 upregulated SOCS2 to facilitate SLC7A11-mediated ferroptosis, whereby suppressing the growth of tumors. This was validated through *in vivo* experiments, which showed that lncRNA-CASC2 overexpression impeded the proliferative ability of GC cells and promoted ferroptosis in nude mice, accompanied by reduced SCL7A11 expression and elevated SOCS2 expression.

**Fig. 9:**
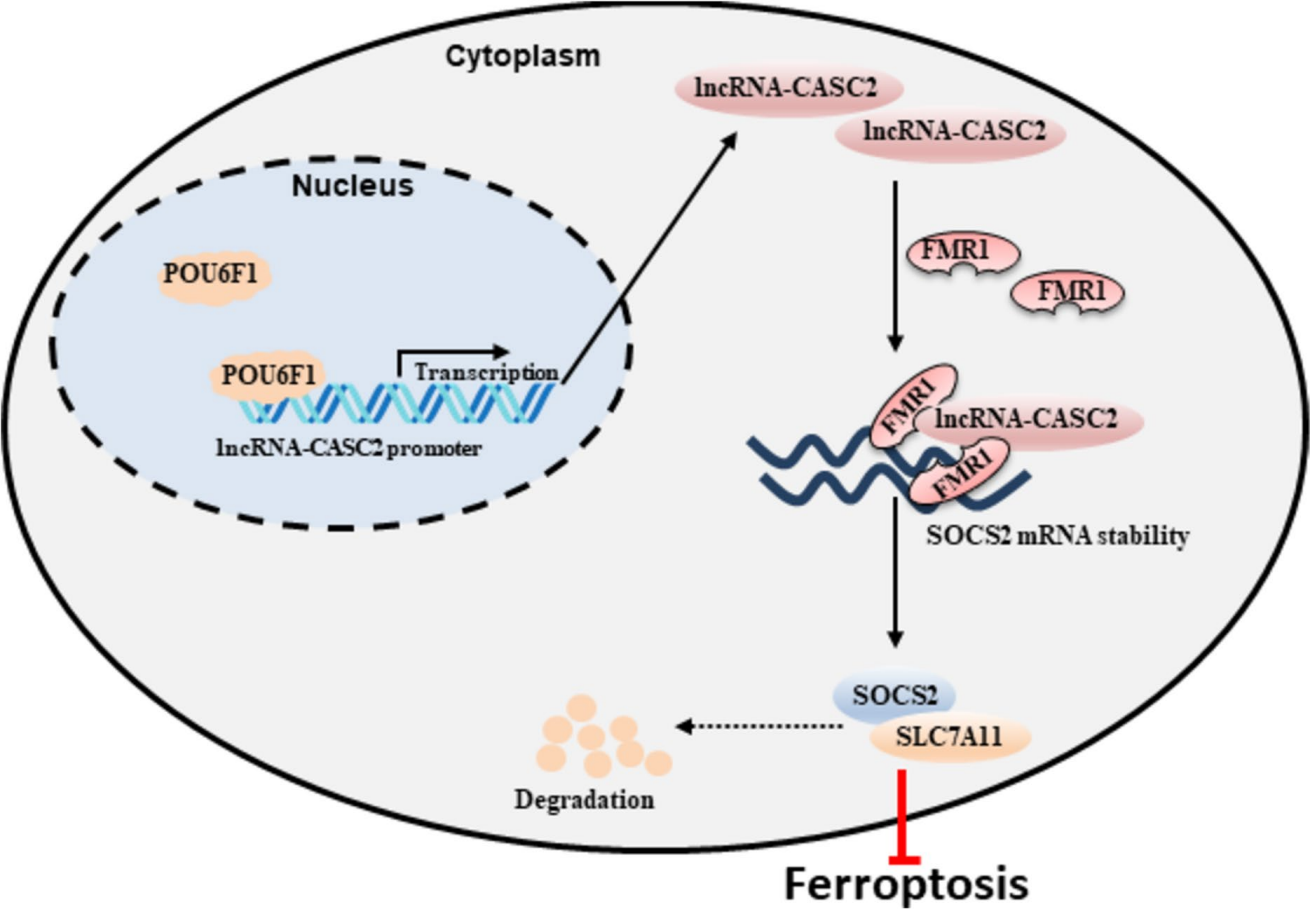
Graphical abstract

In summary, in this paper, a new mechanism mediated by lncRNA-CASC2 to affect ferroptosis was proposed. POU6F1 upregulates the transcription of lncRNA-CASC2, and in turn, lncRNA-CASC2 increases the stability of SOCS2 *via* FMR1, promoting the ubiquitination-dependent degradation of SCL7A11. As a consequence, ferroptosis is boosted to retard the growth of gastric tumors (Fig. 9). These findings suggest the possibility of lncRNA-CASC2 as a putative target for the treatment of GC and also improve our cognition in the pathophysiology of GC. However, more studies are necessary to validate these findings, and problems regarding lncRNA targets remain to be solved before clinical translation.

## Supplementary information

ESM 1 Figure S Overexpression of POU6F1 or lncRNA-CASC2 was achieved in GC cells. (A-B) After cell transfection, POU6F1 and LncRNA CASC2 expressions were detected. (C) AGS or MKN-45 cells were treated with Erastin or both Erastin and overexpression of POU6F1/lncRNA-CASC2, CCK-8 detected the survival rate; (D) AGS or MKN-45 cells were treated with RSL3 or both RSL3 and overexpression of POU6F1/lncRNA-CASC2, and the survival rate was measured by CCK-8.

## Data Availability

The datasets used or analyzed during the current study are available from the corresponding author on reasonable request.
