# Supplementary figures and images for: POU6F1 promotes ferroptosis by increasing lncRNA-CASC2 transcription to regulate SOCS2/SLC7A11 signaling in gastric cancer
Source: Cell Biol Toxicol. 2024 Jan 25;40(1):3. doi: 10.1007/s10565-024-09843-y (PMC10808632; doi:10.1007/s10565-024-09843-y)

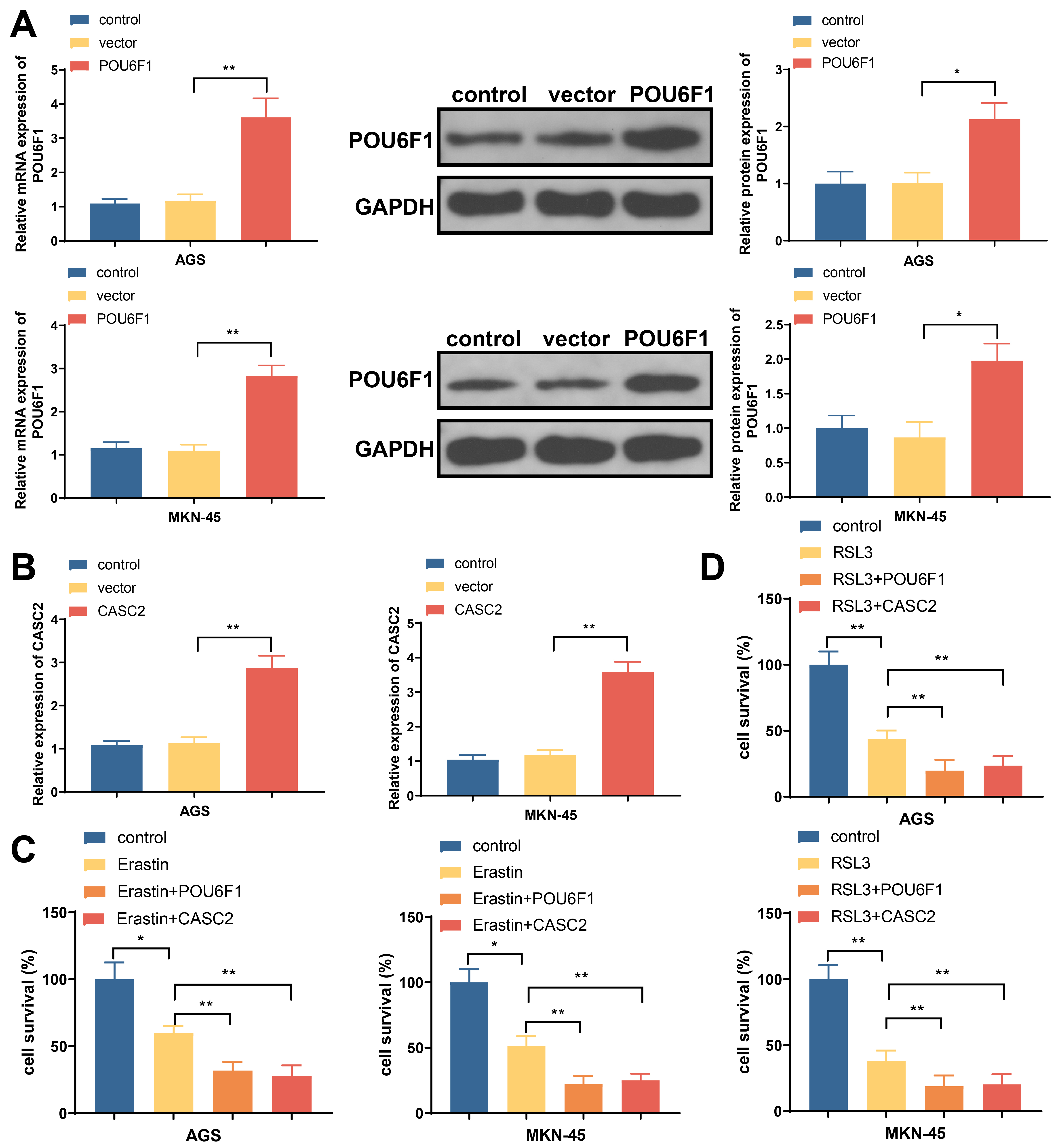

Supplement: Supplementary file 1 — Figure S Overexpression of POU6F1 or lncRNA-CASC2 was achieved in GC cells. (A-B) After cell transfection, POU6F1 and LncRNA CASC2 expressions were detected. (C) AGS or MKN-45 cells were treated with Erastin or both Erastin and overexpression of POU6F1/lncRNA-CASC2, CCK-8 detected the survival rate; (D) AGS or MKN-45 cells were treated with RSL3 or both RSL3 and overexpression of POU6F1/lncRNA-CASC2, and the survival rate was measured by CCK-8. [file 10565_2024_9843_MOESM1_ESM.jpg]
